# Therapeutic Effects of *Hemerocallis citrina* Baroni Extract on Animal Models of Neurodegenerative Diseases Through Serotonin and HLH-30/TFEB-Dependent Mechanisms

**DOI:** 10.3390/ijms26094145

**Published:** 2025-04-27

**Authors:** Jorge H. Fernandes, Marta Daniela Costa, Daniela Vilasboas-Campos, Bruna Ferreira-Lomba, Joana Pereira-Sousa, Qiong Wang, Andreia Teixeira-Castro, Xinmin Liu, Fengzhong Wang, Alberto C. P. Dias, Patrícia Maciel

**Affiliations:** 1 Life and Health Sciences Research Institute (ICVS), School of Medicine, University of Minho, 4710-057 Braga, Portugal; 2 ICVS/3B’s—PT Government Associate Laboratory, 4710-057 Braga, Portugal; 3 Institute of Food Science and Technology, Chinese Academy of Agricultural Sciences, Beijing 100193, China; 4 Institute of Drug Discovery Technology, Ningbo University, Ningbo 315211, China; 5 Centre of Molecular and Environmental Biology (CBMA), Department of Biology, University of Minho, 4710-057 Braga, Portugal

**Keywords:** medicinal plants, neurodegenerative diseases, Machado–Joseph disease/spinocerebellar ataxia type 3, Frontotemporal Dementia with Parkinsonism associated to chromosome 17

## Abstract

*Hemerocallis citrina* is an herbaceous perennial plant used in Asian cuisine and Traditional Chinese Medicine. Here, we tested the therapeutic potential of extracts (HCE30%, HCE50%, and HCN) in vivo, using models of two human genetic neurodegenerative diseases—Machado–Joseph Disease/Spinocerebellar Ataxia type 3 (MJD/SCA3) and Frontotemporal Dementia with Parkinsonism associated to chromosome 17 (FTDP-17). Chronic treatment with HCE30% extract ameliorated the motor deficits typically observed in these models. Interestingly, we found that the effect on the motor phenotype of the MJD/SCA3 model was dependent on serotonergic signaling and on the action of the HLH-30/TFEB transcription factor, known to regulate the cellular response to amino acid starvation, the autophagy and mitophagy pathways, lysosome localization and biogenesis, exocytosis, and mitochondrial biogenesis. Altogether, our findings reinforce the idea that phytochemicals act through the modulation of serotonergic neurotransmission and introduce a novel layer to the HLH-30/TFEB regulatory network. Thus, it also strengthens the use of these pathways as therapeutic targets for protein-related neurodegenerative disorders and confirms the utility of medicinal plants as a source of innovation in the quest for new therapeutic agents.

## 1. Introduction

Neurodegenerative diseases (NDs) are a heterogeneous group of diseases characterized by progressive loss of neurons, many of them associated with the accumulation of misfolded protein and protein aggregates [1]. Developing an effective treatment for NDs is currently a great challenge in medicine, given their increasing prevalence in association with longer life expectancies [2]. Despite extensive research in the field, these highly debilitating diseases remain incurable, necessitating urgent novel therapeutic strategies. Edible plants traditionally used in Asian cuisines are also often used in Traditional Medicine and have garnered increasing attention as functional foods, as they often contain bioactive compounds that may confer health benefits beyond basic nutrition. Owing to their chemical diversity and complexity, plant-derived products emerge as a very interesting source for novel bioactive compounds [3]. Several studies using in vitro and in vivo models of NDs have demonstrated the ability of plant-based products to successfully target pathological events associated with NDs, namely by reducing the abnormal accumulation of disease-specific misfolded and aggregated proteins (Reviewed in [3]). Among the complex pathologies of these diseases, oxidative stress is another hallmark frequently modulated by treatments derived from plants, as we previously demonstrated for *Hyptis* spp. extracts and Rapeseed Pomace, a waste product from edible oil production from *Brassica napus* [4,5].

*Hemerocallis citrina* Baroni is a herbaceous perennial plant from the Liliaceae family, native to East Asia, commonly known as the “daylily flower” or “yellow flower vegetable”, and with widespread use in Asian cuisine. It is also listed in the “Compendium of Materia Medica” for its bioactive properties, historically used for its sedative, sleep-promoting, and febrifuge activities [6]. Its flowers, the edible part of the plant, are rich in flavonoids, namely Rutin and phenylpropanoids, such as quinic acid esters, especially those conjugated with caffeic, p-hydroxycinnamic, and ferulic acid and their glucosides [7]. Several studies have been conducted on the pharmacological potential of *H. citrina* Baroni, with promising results. *H. citrina* Baroni extracts prevented injury induced by glutamate and corticosterone and increased serotonin release in PC12 cells [8]. In vivo studies showed an antidepressant-like effect on rodents, mainly with an impact on the monoaminergic system, elevating serotonin, noradrenaline, and dopamine levels [6]. In previous studies with nematode and mouse models of Machado–Joseph disease (MJD) or Spinocerebellar ataxia type 3 (SCA3)—recognized as the most common autosomal-dominant form of genetic ataxia worldwide—it was demonstrated that modifying the serotonergic signaling pathways led to improvements in key aspects of this ND, namely reducing mutant protein aggregation [9,10]. Given that *H. citrina* extracts have already demonstrated the ability to modulate neurotransmitter signaling, we hypothesized that they could have a beneficial impact on NDs by modulating serotonergic or dopaminergic signaling.

In this work, taking advantage of the nematode model, we aimed to explore the therapeutic potential of *H. citrina* Baroni flower extracts in genetic models of MJD/SCA3—a genetically inherited spinocerebellar ataxia caused by an abnormal expansion of a CAG in the *ATXN3* gene—and frontotemporal dementia with parkinsonism associated with chromosome 17 (FTDP-17)—a genetically determined form of dementia caused by mutation in the microtubule-associated protein tau (*MAPT*) gene [11,12,13,14]. Additionally, using genetic tools and reporter strains, we expect to dissect the mechanism(s) driving the beneficial effect of these extracts.

## 2. Results

### 2.1. H. citrina Baroni Ethanolic Extracts Ameliorate Motor Deficits in the Model of MJD/SCA3, Independently of Effects on mATXN3 Neuronal Aggregation

**Figure 1:**
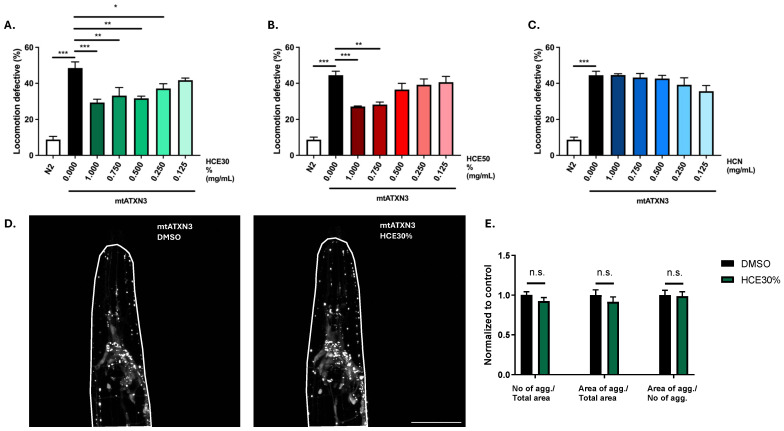
*H. citrina* Baroni 30% ethanolic extract (HCE30%) improves MJD motor deficits independent of ATXN3 aggregation in neurons. (**A**). HCE30%, (**B**). HCE50%, and (**C**). HCN effect on locomotion deficits of mutant ataxin-3 (mtATXN3)-expressing animals (0.125–1.000 mg/mL) compared to animals treated with vehicle only (DMSO 1%) and N2 WT animals. Bars represent the mean percentage of animals considered as locomotion defective in a motility assay ± SEM for 3 independent assays, at least 50 animals per condition, per assay (total number of animals = 150). *p* ≤ 0.05 (*); *p* ≤ 0.01 (**); *p* ≤ 0.001 (***). (**D**). Confocal imaging of the head of AT3q130 animals treated with vehicle (DMSO 1%) or HCE30% (1 mg/mL). Pictures are representative of three independent experiments. (**E**). Quantification of the number of mutant ataxin-3 aggregates normalized per total area, area of aggregates per total area, and area of aggregates per total number of aggregates. Bars represent the mean, normalized to vehicle-treated ± SEM of at least 10 animals per condition per experiment. *p* < 0.05; no significant differences (n.s.). Scale bar 50 µm.

Treatment with *H. citrina* extracts did not present any toxicity (Supplementary Figure S1). While HCN did not have any effect on the model’s motility, HCE50% treatment resulted in improvement of the MJD/SCA3 model’s motility at the highest concentrations (1 mg/mL and 0.75 mg/mL), and HCE30% was effective down to 0.25 mg/mL, suggesting a higher potency and a broader therapeutic window (Figure 1A–C). When treatment with the most efficient dosage of HCE30% (1 mg/mL) was prolonged for 6 days, with the extract being renewed on day 5 and day 6 to exclude progeny, treated animals also performed better when compared to vehicle-treated ones, with no observable undesirable effect (Supplementary Figure S2). Likewise, to explore the durability of the effect, we performed an Off-Drug assay, where animals were treated for a period of 4 days, after which they were removed, washed, and treated with a vehicle for 2 days. The motility assessment revealed that discontinuation of the treatment resulted in the loss of the beneficial effect (Supplementary Figure S2).

One hallmark of MJD is the formation of mtATXN3 aggregates within specific regions of patients’ brains. This pathological aspect of the disease is also present in the *C. elegans* model and can be quantified. Treatment with HCE30% at 1 mg/mL did not alter mutant ATXN3 aggregation dynamics in the model at the onset of adulthood. Confocal imaging showed that treated animals did not have a different number of neuronal mtATXN3 aggregates per total head area, nor a distinct area occupied by the aggregates. The size of the aggregates was also not affected (Figure 1D,E).

### 2.2. H. citrina Baroni Ethanolic Extract Ameliorates Motor Deficits in a Model of FTDP-17

**Figure 2:**
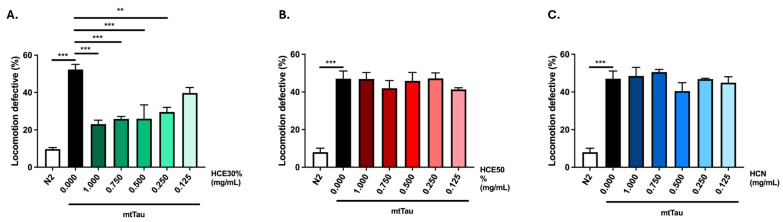
*H. citrina* Baroni 30% ethanolic extract (HCE30%) improves motor deficits in an FTDP-17 *C. elegans* model expressing mutant Tau protein (mtTau). (**A**). HCE30%, (**B**). HCE50%, and (**C**). HCN effect on locomotion deficits of mtTau (0.125–1.000 mg/mL) compared to animals treated with vehicle only (DMSO 1%) and N2 WT animals. Bars represent the mean percentage of animals considered as locomotion defective in a motility assay ± SEM for 3 independent assays, with at least 50 animals per condition per assay (total number of animals = 150). *p* ≤ 0.01 (**); *p* ≤ 0.001 (***).

To assess the potential therapeutic benefits of *H. citrina* Baroni extracts for other neurodegenerative diseases, a *C. elegans* FTDP-17 model, expressing a mutant (V337M) Tau protein (V337M, here designated mtTau), was chronically treated with the three *H. citrina* Baroni extracts (Figure 2A–C) [12]. HCE30% treatment was the only condition to significantly reduce locomotion deficits of the model, in a dose-dependent manner. The comparison between animals treated with HCE30% and DMSO1% revealed a significantly improved motor phenotype of HCE30%-treated animals with 1.000 mg/mL, 0.750 mg/mL, 0.500 mg/mL, and 0.250 mg/mL. Due to their broader therapeutic potential in two distinct neurodegenerative conditions, HCE30% (hereinafter referred to as HCE) was further studied, and all experiments were performed at a dosage of 1.000 mg/mL.

### 2.3. Phytochemical Composition of the Extracts

**Figure 3:**
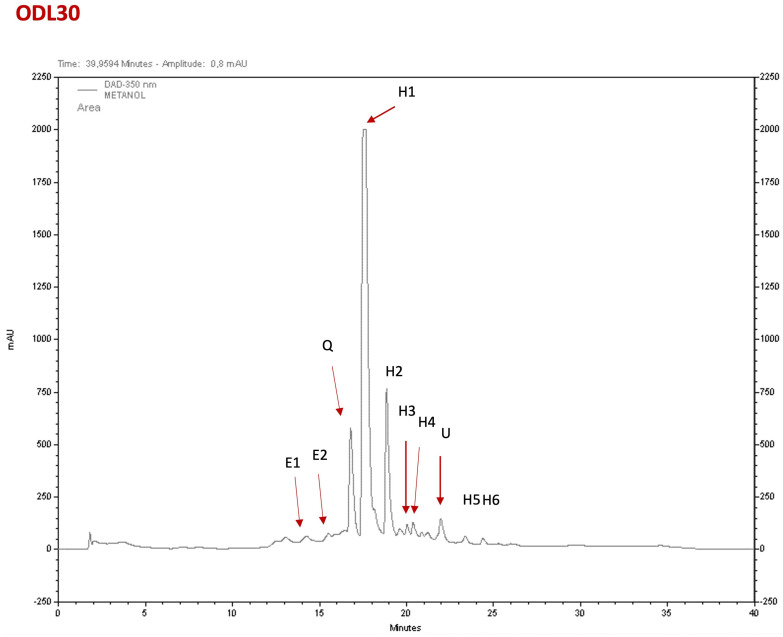
The phytochemical profile of HCE30%. The chromatographic analysis revealed that HCE30% was mainly composed of (E1) epicatechin and (E2) epicatechin derivative, (Q) quercetin glucoside, and up to 70% of (H1–H6) hydroxycinnamic acids. U, unknown compound with no exact correspondence to retention time. Quantification can be found in Supplementary Table S2.

The major constituents of the *H. citrina* Baroni extracts (HCE) were hydroxycinnamic acids and flavonoids, based on their characteristic UV spectrum profile [15]. HCE30% was the fraction that showed more relevant pharmacological activities. A typical chromatogram of HCE30% is shown in Figure 3, and the identification and quantification of the compounds are indicated in Supplementary Table S1. Its composition is constituted by hydroxycinnamic acids (up to 70%), with minor amounts of epicatechins and a quercetin glucoside derivative. This HPLC profile and composition are similar to those previously reported with another HCE extract used before [8]. 

### 2.4. HCE Effect Was Dependent on Serotonergic but Not Dopaminergic Signaling in the MJD/SCA3 Model

**Figure 4:**
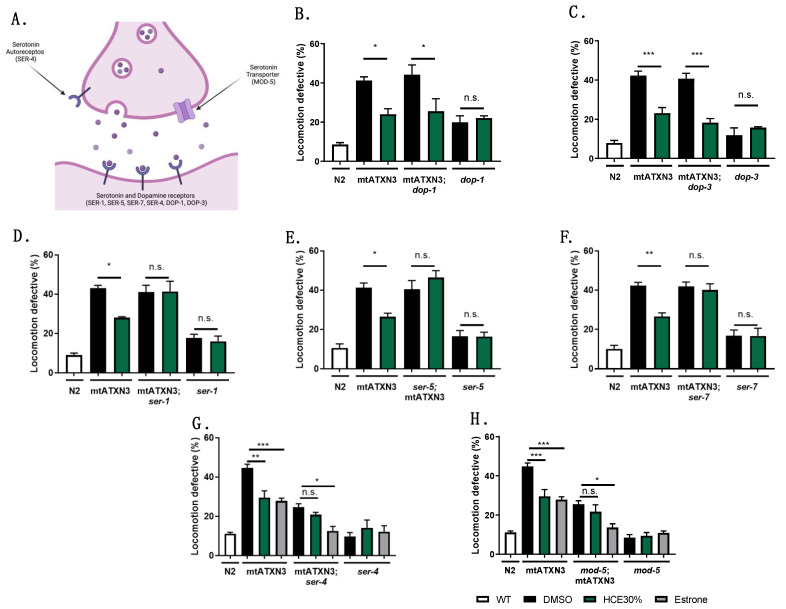
Motor phenotype amelioration of *C. elegans* model of MJD/SCA3 by *H. citrina* Baroni 30% ethanolic extract is dependent on serotonergic but not dopaminergic signaling. (**A**). Schematic representation of a synapse with dopaminergic and serotonergic receptors. HCE is effective in reducing motor deficits in the mtATXN3 background when (**B**). dop-1 and (**C**). dop-3 dopamine receptors are ablated. Knock-down of serotonergic receptors (**D**). Ser-1, (**E**). Ser-5, and (**F**). Ser-7 resulted in total loss of the motor phenotype improvement provoked by HCE treatment. Ablation of serotonin autoreceptor (**G**). Ser-4 and transporter (**H**). mod-5 improved the motor deficits displayed by the MJD nematode model, an effect that was further extended by treatment with estrone (which has a mode of action independent of serotonergic signaling) while HCE treatment did not have an additive effect, suggesting that the intervention is acting in the same pathway as the genetic modification. Statistics: (**B**–**H**). One-way ANOVA followed by post-hoc Tukey HSD test for multiple comparisons. Bars represent mean ± SEM. n = 4; *p* ≤ 0.05 (*); *p* ≤ 0.01 (**); *p* ≤ 0.001 (***); no significant differences (n.s.).

Other *H. citrina* Baroni extracts have been shown to modulate serotonergic and dopaminergic systems in rodent models [6,16]. Similarly, our team has previously demonstrated the modulation of the serotonergic system by citalopram or of both serotonergic and dopaminergic systems by aripiprazole chronic treatments, ameliorating the motor dysfunction in the mtATXN3-expressing *C. elegans* model [10,17,18]. Therefore, we examined whether the dopaminergic and serotoninergic systems in *C. elegans* were necessary for the previously described beneficial impact of HCE by ablating their main receptors in the mtATXN3 genetic background (Figure 4A).

Ablation of both DOP-1 (D1-like) and DOP-3 (D2-like) receptors did not affect HCE’s effect on the locomotion of mtATXN3-expressing worms (Figure 4B,C). In contrast, ablation of the serotonin receptors, SER-1 (5-HT2A-like) (*p* = 1.000), SER-5 (5-HT6-like) (*p* = 0.774), or SER-7 (5-HT7-like) (*p* = 0.999), led to a complete loss of HCE effect (Figure 4D–F), indicating that serotonergic, but not dopaminergic, signaling is necessary for HCE efficacy.

As several post-synaptic serotonin receptors were necessary for the HCE effect in the MJD/SCA3 model, we hypothesized that HCE might be increasing the availability of serotonin in the synaptic cleft. Therefore, we evaluated the HCE action dependency on the SER-4 (5-HT1A-like) receptor and MOD-5 (SERT-like) transporter involved in the reuptake of serotonin from the synaptic cleft [10,19,20]. Both proteins are involved in negative feedback mechanisms to reduce the amounts of serotonin available in the synaptic cleft. As we previously demonstrated, ablation of SER-4 or MOD-5 per se significantly improves the motor behavior of the MJD/SCA3 model [10]. Interestingly, HCE chronic treatment in the MJD/SCA3 model, knockout for the SER-4 autoreceptor or the MOD-5 transporter, did not have an additive beneficial effect, as observed with estrone treatment. Estrone improves the model’s motor phenotype, independently from the serotonergic system [10] (Figure 4G,H). Overall, these findings suggest that the effect of HCE treatment in the *C. elegans* model of MJD/SCA3 is dependent on a broad action of the extract on the serotonergic system.

### 2.5. HCE Treatment Promotes HLH-30/TFEB Nuclear Translocation

**Figure 5:**
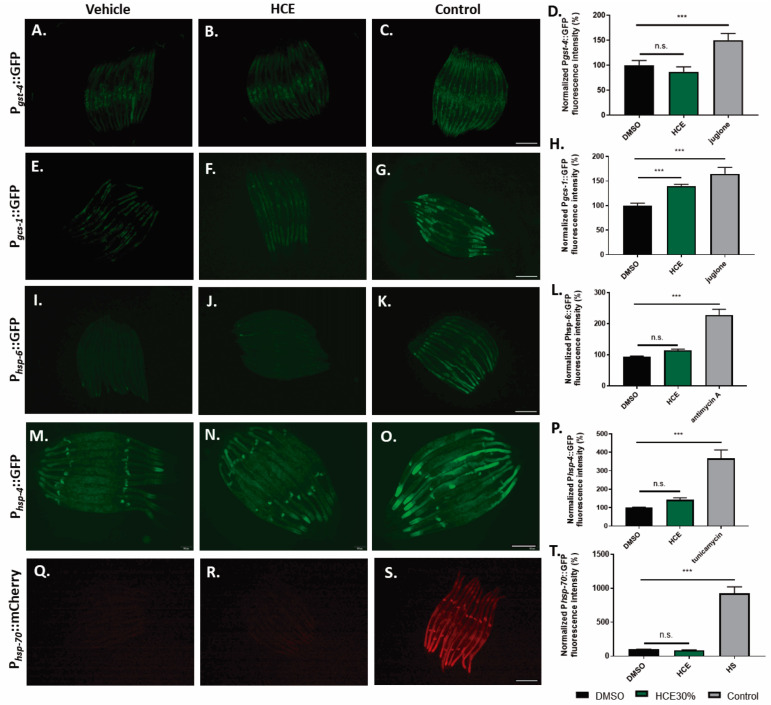
HCE treatment did not induce the antioxidant response, the ER or Mitochondrial Unfolded Protein Response, or the Heat Shock Response pathways. Fluorescence images of P*gst-4*::GFP animals treated with (**A**). vehicle (DMSO 1%), (**B**). HCE (1 mg/mL), and (**C**). vehicle-treated, followed by juglone treatment as a positive control. Scale bar = 300 µm. Graphical results shown in (**D**). represent the fluorescence levels, normalized for the control. Fluorescence images of P*gcs-1*::GFP animals treated with (**E**). vehicle (DMSO 1%), (**F**). HCE (1 mg/mL), and (**G**). vehicle-treated, followed by juglone treatment as a positive control. Scale bar = 300 µm. Graphical results shown in (**H**). represent the fluorescence levels, normalized for the control. Fluorescence images of P*hsp-6*::GFP animals treated with (**I**). vehicle (DMSO 1%), (**J**). HCE (1 mg/mL), and (**K**). vehicle-treated, followed by antimycin A treatment as a positive control. Scale bar = 300 µm. Graphical results shown in (**L**). represent the fluorescence levels, normalized for the control. Fluorescence images of P*hsp-4*::GFP animals treated with (**M**). vehicle (DMSO 1%), (**N**). HCE (1 mg/mL), and (**O**). vehicle-treated, followed by tunicamycin treatment as a positive control. Scale bar = 200 µm. Graphical results shown in (**P**). represent the fluorescence levels, normalized for the control. Fluorescence images of P*hsp-70*::mCherry animals treated with (**Q**). vehicle (DMSO 1%), (**R**). HCE (1 mg/mL), and (**S**). vehicle-treated, followed by heat-shock at 33 °C for 60 min, followed by a period of recovery of 7 h and treatment as a positive control. Scale bar = 300 µm. Graphical results shown in (**T**) represent the fluorescence levels, normalized for the control. Bars represent mean ± SEM. n = 15 *p* ≤ 0.001 (***); no significant differences (n.s.).

To gain mechanistic insight into the action of the extract on several endogenous cellular stress responses, *C. elegans* transcriptional reporter strains were used to quantify the stress-response activation upon HCE treatment. We investigated the effect of HCE treatment on the expression of antioxidant-related genes, namely the *gst-4* and *gcs-1* genes, encoding, respectively, the drug-metabolizing enzyme glutathione S-transferase 4 (GST-4) and the Phase-II detoxification enzyme gamma-glutamylcysteine synthetase (GCS-1), both involved in the glutathione cycle. When chronically treated with HCE extract, no impact was observed regarding the promoter activity of *gst-4* (Figure 5A–D), while the *gcs-1* transcriptional reporter strain showed a mild increase in GFP fluorescence compared to the untreated control (Figure 5E–H). However, the fluorescence pattern observed did not match the one seen for the positive control (acute juglone treatment), raising doubts about the biological significance of this result.

To investigate the hypothesis of HCE acting by the modulation of protein folding and homeostasis, we used transcriptional reporter strains for the Unfolded Mitochondrial Protein Response (UPRmt):P*hsp-6*::GFP (Figure 5I,J), for the Unfolded Protein Response of the endoplasmic reticulum (UPRER): P*hsp-4*::GFP (Figure 5M–P), and for the Heat Shock Response (HSR): P*hsp-70*::mCherry (Figure 5J–T). Our results showed that HCE treatment had no impact on the activity of any of the promoters.

**Figure 6:**
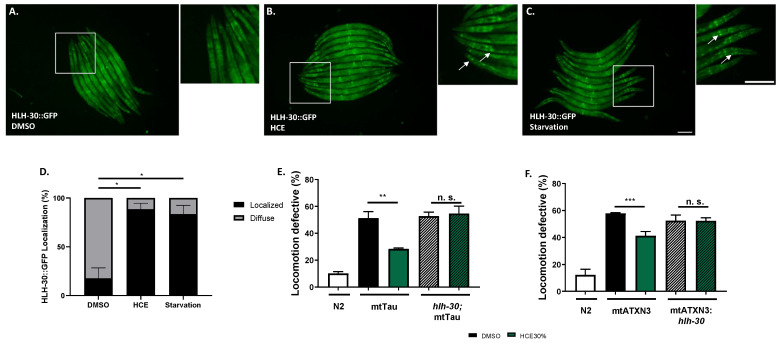
HCE treatment induces HLH-30 nuclear translocationFluorescence images of (**A**). vehicle (DMSO 1%), (**B**). HCE (1.000 mg/mL), and (**C**). vehicle-treated, followed by a 30-min starvation period, P*_hlh-30_*::HLH-30::GFP animals. Arrows point to examples of nuclear translocation. Scale bar = 300 μm. Graphical results shown in (**D**). represent the proportion of animals that presented nuclear localization of the HLH-30 transcription factor as displayed by the formation of clear GFP puncta. Treatment with HCE resulted in nuclear translocation of the transcription factor to levels similar to starvation. Pearson’s Chi-square test and post-hoc pairwise comparisons using Z-tests were conducted to compare column proportions; *p* ≤ 0.05 (*). HLH-30/TFEB transcription factor is necessary for the HCE effect and is dependent on serotonergic signaling. Ablation of the hlh-30 in the (**E**) mtTau and (**F**) mtATXN3 background resulted in the loss of the motor phenotype amelioration. Bars represent mean ± SEM. n = 4; *p* ≤ 0.05 (*); *p* ≤ 0.01 (**); *p* ≤ 0.001 (***); no significant differences (n.s.).

We also assessed the ability of HCE to activate the HLH-30, the nematode orthologue of TFEB, a mammalian transcription factor that regulates the expression of several autophagy-related genes, among others [21]. When activated, HLH-30 is translocated from the cytoplasm to the nucleus, where it promotes the transcription of autophagy-related genes, such as *atg-18*, *lgg-1*, and *sqst-1*; components of the insulin signaling pathway as *ins-11*, *sgk-1*, and *dct-1*, which are known longevity regulators; host-defense signaling pathways, namely *kgb-1*, *nsy-1*, and *mdl-1*; genes with antimicrobial activity such as those encoding C-type lectins, antimicrobial peptides, and ferritin, as well as detoxifying enzymes such as *fmo-2* [21,22,23]. Notably, with HCE treatment, we observed an induction of HLH-30::GFP nuclear translocation, similar to animals in starvation conditions (Figure 6A–D). This suggests that HCE treatment is inducing HLH-30/TFEB-dependent responses in the nematode. HLH-30 has been shown to promote the mobilization of lipid droplets during stress by promoting the expression of acid lipases, capable of breaking down lipid droplets [24]. To further understand the impact of HLH-30 on metabolic adaptation, we went on to address the lipid dynamics of the animal upon treatment and assess potential effects on the consumption of neutral lipids from lipid droplets. To this end, we performed staining using BODIPY 493/503 upon treatment with vehicle and HCE (Supplementary Figure S3A,B). We observed no impact on the proportion of the animals’ body area stained with BODIPY 493/503 (Supplementary Figure S3C), nor on the average size of lipid droplets (Supplementary Figure S3D). We next sought to explore whether the beneficial effect of HCE in the FTDP-17 and MJD/SCA3 *C. elegans* models was dependent on the presence of HLH-30. Upon ablation of HLH-30, HCE treatment lost the beneficial effect in the mtATXN3 (Figure 6E) and mtTau genetic backgrounds (Figure 6F), suggesting that HLH-30 is indeed necessary for the effect of this herbal extract.

### 2.6. HLH-30/TFEB Activation by HCE Treatment Requires Serotonergic Signaling

**Figure 7:**
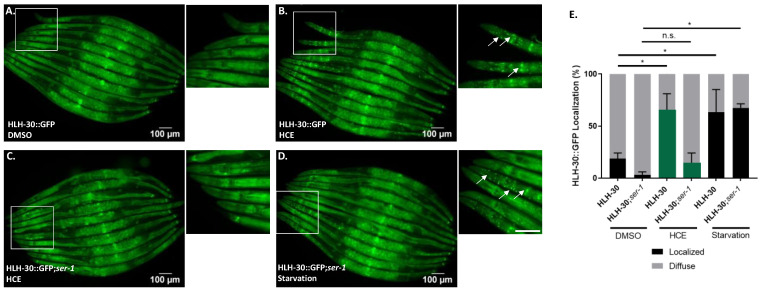
HCE activation of HLH-30 depends on the SER-1 serotonin receptor. Brightfield and fluorescence images of (**A**). *P_hlh-30_*::HLH-30::GFP animals treated with vehicle (DMSO 1%), (**B**). HCE (**C**). *P_hlh-30_*::HLH-30::GFP; *ser-1* animals treated with HCE and (**D**). *P_hlh-30_*::HLH-30::GFP; *ser-1* animals treated with vehicle followed by a 30-min starvation period. Arrows point to examples of nuclear translocation. Scale bar = 100 μm. Graphical results shown in (**E**). represent the proportion of animals that presented nuclear localization of the HLH-30 transcription factor as displayed by the formation of clear GFP puncta. The green bars are representative of HCE treatment. Treatment with HCE30% resulted in nuclear translocation of the transcription factor in levels similar to starvation; *p* ≤ 0.05 (*); no significant differences (n.s.).

Considering the findings indicating that HCE effects in MJD/SCA3 depended on serotonergic signaling and that this extract induced HLH-30/TFEB nuclear translocation (Figure 4 and Figure 6), we next investigated whether HLH-30/TFEB activation was also controlled by serotonergic signaling. In the double mutant resulting from the crossing between the HLH-30::GFP *C. elegans* reporter strain and a strain lacking the SER-1 receptor, we observed a significantly reduced HLH-30 nuclear localization upon HCE treatment compared to the parental strain (Figure 7A–E). This result indicated that, in HCE-treated animals, HLH-30 nuclear translocation is dependent on serotonergic signaling. Intriguingly, no impact on HLH-30 nuclear translocation was observed in the starved animals, suggesting that starvation activates HLH-30 through a different pathway and independently from serotonergic signaling. This indicates that HCE depends on the serotonergic system to elicit the activation of the HLH-30 transcription factor.

### 2.7. HCE Effects May Be Mediated by Quercetin or Its Glucoside Derivatives

Considering the findings indicating that HCE has a positive impact on the motor defects displayed by *C. elegans* models of NDs, we sought to find the molecules potentially responsible for its action within the extract. Having in mind the HPLC fingerprint of HCE extract that revealed the presence of hydroxycinnamic acid derivatives, as well as quercetin glucoside derivatives, different concentrations of (1) chlorogenic acid (a hydroxycinnamic acid, an ester of caffeic acid and quinic acid, previously described to be present in *H. citrina*), (2) Quercetin (a flavonoid) and isoquercitrin (a quercetin glucoside derivative), also previously associated with activities of *H. citrina*, and (3) Gallic acid, a phenolic acid, commonly found in the flowers of *H. citrina*, whose chemical properties are consistent with those captured by our extraction method, were tested on the motor phenotype of AT3q130 animals.

**Figure 8:**
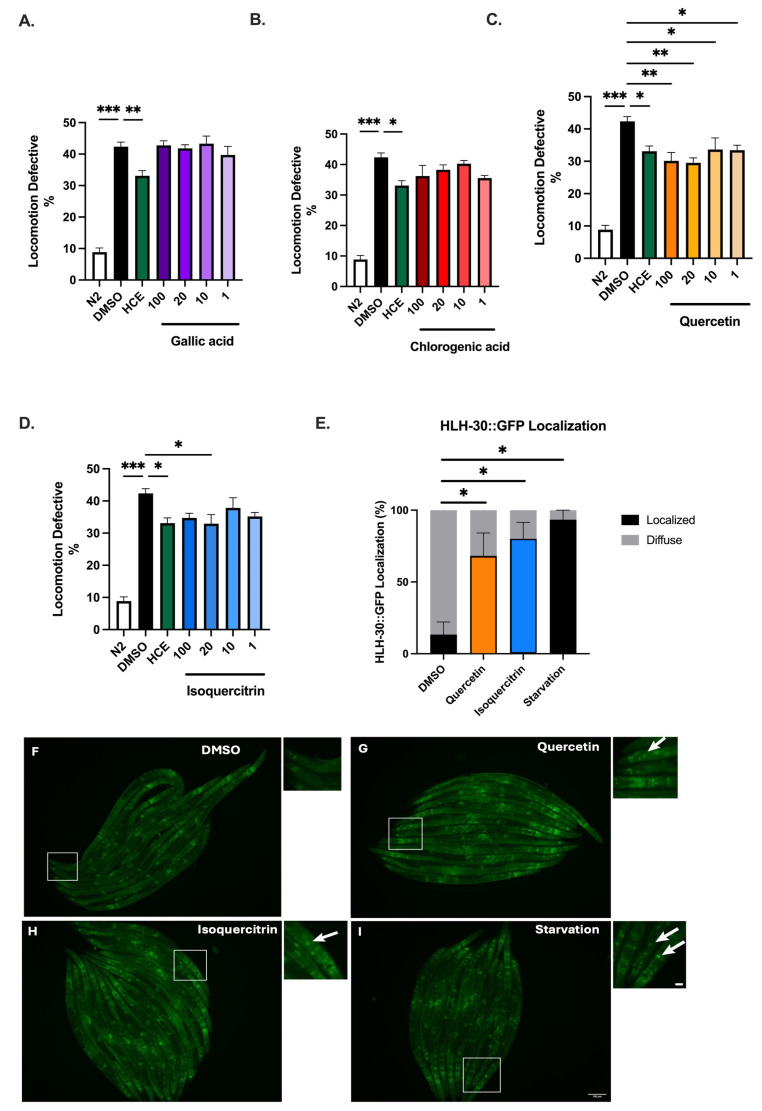

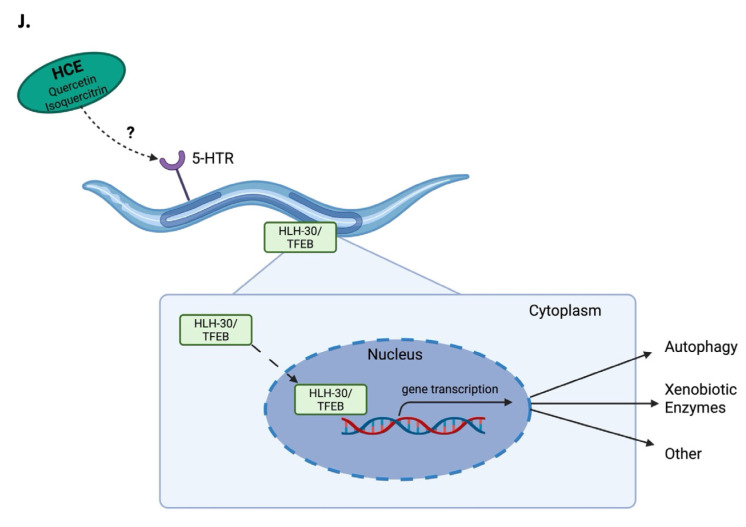
Quercetin and isoquercitrin recapitulate the beneficial effects of HCE in animal models of MJD/SCA3. Effect of (**A**). Gallic Acid, (**B**). Chlorogenic Acid, (**C**). Quercetin, and (**D**). Isoquercitrin on locomotion deficits of mutant ataxin-3 (mtATXN3)-expressing animals (100–1 μM) compared to animals treated with vehicle only (DMSO 1%) and N2 WT DMSO-treated animals. Bars represent the mean percentage of animals considered as locomotion defective in a motility assay ± SEM for three independent assays, with at least 50 animals per condition, per assay (total number of animals = 150). *p* ≤ 0.05 (*); *p* ≤ 0.01 (**); *p* ≤ 0.001 (***). (**E**) Proportion of Phlh-30::HLH-30::GFP animals that presented nuclear localization of the HLH-30 transcription factor as displayed by the formation of clear GFP puncta upon treatment with (DMSO 1%), Quercetin (100 μM), and Isoquercitrin (μM). Confocal microscopy images of Phlh-30::HLH-30::GFP animals treated with (**F**). vehicle (DMSO 1%), (**G**). Quercetin (100 μM), (**H**). Isoquercitrin (20 μM), and (**I**). animals treated with vehicle followed by a 30-min starvation period. Arrows point to examples of nuclear translocation. Scale bar: 100 μm. Three independent assays were performed, with at least 10 animals per condition, per assay (total number of animals = 30), *p* ≤ 0.05 (*). (**J**). Schematic representation of the serotonin and HLH-30/TFEB-dependent mode of action of HCE in *C. elegans*. Pictures are representative of three independent experiments.

Unlike gallic (Figure 8A) and chlorogenic acids (Figure 8B), both quercetin (Figure 8C) and isoquercitrin (Figure 8D) improved the motor phenotype of mtATXN3-expressing animals. Moreover, treatment with both quercetin and isoquercitrin (Figure 8E–I) also leads to the nuclear translocation of HLH-30/TFEB, suggesting that these two molecules could mediate the effects of HCE.

## 3. Discussion

Early diagnosis and timely interventions are possible for genetically determined neurodegenerative diseases. Nevertheless, the absence of an effective treatment hinders a more successful patient outcome, thus remaining an urgent medical need. In this study, we showed that a chronically applied extract derived from an Asian edible plant, *H. citrina* Baroni, improved, in a dose-dependent manner, the motor phenotypes of two animal models of genetically determined NDs: MJD/SCA3, caused by mutant ataxin-3, and FTDP-17, determined by a mutant form of the Tau protein. Despite the different genetic origins, these diseases exhibit shared clinical and mechanistic characteristics, namely aggregation of the disease-causing proteins in the brain, progressive neuronal loss, and impaired movement (which in FTDP-17 is associated with a very relevant cognitive impairment). By demonstrating the therapeutic potential of extracts from *H. citrina* Baroni for these diseases, we support the concept of therapeutically targeting pathogenic events shared between neurodegenerative conditions. *C. elegans* serves as a valuable model for studying neurodegeneration due to its well-characterized nervous system and conserved orthology with mammalians [25]. Moreover, it is important to note that the organization of the serotonergic system remains similar; this species shares the biosynthetic pathway and vesicle loading and reuptake machinery with vertebrates [26]. However, we acknowledge its physiological differences, which may limit the direct translation of our findings to human neurodegenerative disorders. For example, *C. elegans* lacks a blood-brain barrier and has a simpler neuronal network, which can influence pharmacokinetics and the neuroprotective effects found. Further validation using mammalian models is essential to confirm its clinical relevance.

It was demonstrated in other studies that extracts from *H. citrina* Baroni modulated the levels of serotonin and other neurotransmitters in the frontal cortex and hippocampus of mice [6]. Similarly, several components of the *H. citrina* Baroni extracts were described to affect the serotonergic system: (1) Rutin, a quercetin glucoside derivative, increased serotonin in the hippocampus of a rat model of diabetes; (2) chlorogenic acid, a hydroxycinnamic acid, promoted serotonin release through enhancing synapsin I expression; (3) quercetin has been suggested to bind to 5-HT_1_AR, inhibiting the binding of serotonin; and (4) kaempferol, which is often acylated with hydroxycinnamic acid, inhibited the 5-HT_3_AR [27,28,29,30]. Our phytochemical analysis revealed the presence of several hydroxycinnamic acids and quercetin derivatives. We further tested quercetin and isoquercitrin, a glucoside derivative, in the animal model of MJD/SCA3 and found that treatment with both compounds resulted in improvement of the motor phenotype of this model, suggesting that they could be the main therapeutic actors of HCE. We further confirmed the link between *H. citrina* Baroni extract and the serotonergic system, showing the dependency on serotonergic, but not dopaminergic, receptors for the neuroprotective effect observed in the MJD/SCA3 transgenic model. Manipulating the serotonergic system in mouse models of MJD/SCA3 has been proven to be beneficial in previous studies performed by our team. Citalopram and befiradol chronic treatments specifically targeted the serotonergic system, resulting in an effective treatment, underlining it as a key therapeutic strategy [10,31]. It is of interest that HCE can also act through this mechanism.

We also pinpointed the HLH-30/TFEB transcription factor as another key downstream mediator of the impact of the *H. citrina* Baroni ethanolic extract on MJD. TFEB is a member of the microphthalmia/Transcription Factor E (MiT/TFE) family and also a master regulator of the autophagy-lysosome pathway (Reviewed in [32]) . It is key for stress resistance and elimination of protein aggregates via induction of transcription of genes involved in autophagy, lysosomal biosynthesis, both endo- and exocytosis, and membrane repair (Reviewed in [32]). TFEB dysregulation has been reported in several neurodegenerative diseases, mainly by reduced nuclear localization, such as in the postmortem brains of Alzheimer’s disease, Parkinson’s disease, and amyotrophic lateral sclerosis patients [33,34,35]. Whilst both citalopram and befiradol, serotonergic system-targeting drugs previously shown to be effective in improving the motor phenotype of the mtATXN3 model, had a greater impact on motility than HCE, this study brings important novelty, as it was the first time that serotonin-dependent TFEB activation was observed. Furthermore, TFEB activation as a therapeutic strategy for NDs has recently received attention, with several molecules being tested on neurodegenerative disease models (Reviewed in [34]).

The ortholog of the mammalian TFEB in *C. elegans*, HLH-30, has been conservatively identified as a transcriptional regulator of autophagy [21]. However, several other pathways are also controlled by HLH-30/TFEB in the nematode. For example, HLH-30/TFEB controls the mobilization of lipids under conditions of nutrient starvation [36,37,38]. Neuronal HLH-30/TFEB induces peripheral mitochondrial fragmentation in response to heat stress and regulates the insulin signaling pathway, requiring DAF-16/FOXO, one of the most well-described longevity mechanisms in [39]. A product of natural origin, a benzocoumarin, was also found to induce mitophagy through HLH-30 and DAF-12 activation, without increasing macroautophagy levels, showing a beneficial impact in nematode models of proteinopathies [40]. Lastly, the transcription factor also controls the expression of xenobiotic enzymes, such as the detoxification enzyme flavin-containing monooxygenase-2 (FMO-2), and other proteins with antimicrobial action [23]. FMO-2 overexpression led to an increase in lifespan and health span in multiple lifespan-extending paradigms and improved proteostasis. Furthermore, the impact of FMO-2 on longevity is conserved in mammals [22,41,42]. However, it remains unclear which downstream mechanisms are responsible for the impact of HCE on the models of NDs.

Mechanistically, this study unveiled a previously unrecognized action for *H. citrina* Baroni, identifying the role of HLH-30/TFEB as neuroprotective following activation by the extract. Our results converge with those of other studies using different extracts of *H. citrina* that have identified both quercetin and isoquercitrin as potential bioactive components with neuroprotective activity. Moreover, these studies pointed to the PI3K/AKT pathway as the effector pathway in neurodegeneration [43,44]. This pathway has been described to influence TFEB phosphorylation status and consequently its activity [45].

Our results also revealed that the activation of HLH-30 happens downstream of the serotonergic signaling, as the absence of serotonin receptors abolished HLH-30 activation upon HCE treatment. It is possible that serotonergic transmission may be influencing the non-neuronal subcellular localization and HLH-30/TFEB activity by altering the phosphorylation state of this transcription factor or the activity of upstream regulators of HLH-30/TFEB expression and that this has indirect beneficial action in the neurons through effects other than reduction of aggregation, which could include effects on mitochondria or metabolism, among others. Given the pleiotropic action of HLH-30, future multi-omics studies would be necessary to further detail the HLH-30-dependent molecular events implicated in the impact of *H. citrina* Baroni extracts.

In summary, this work demonstrates the potential of *H. citrina* Baroni*,* an edible plant popularly used in China, as a source for novel compounds with therapeutic potential for tackling pathological events shared by different neurodegenerative diseases. Treatment with an *H. citrina* Baroni ethanolic extract improved a key phenotype of models of MJD/SCA3 and FTDP-17, two NDs with genetically different origins. We identified its mode of action as targeting a possible novel pathway that engages serotonergic signaling and subsequently activates HLH-30, an orthologue of the mammalian TFEB. This underscores the significance of these mechanisms for the future development of novel therapies for neurodegenerative diseases.

## 4. Materials and Methods

### 4.1. Extract Preparation

*H. citrina* Baroni flowers were collected in Qidong County, Hunan Province (China), and a voucher specimen was deposited at the Biology Department at the University of Minho with the reference DB.014.2022. It was extracted three times with 20 volumes of 80% ethanol for 1 h each. The resulting extracts were combined and evaporated under reduced pressure in a rotary vacuum evaporator. After drying, the extract was resuspended in water and re-extracted three times with different solvents: petroleum ether, ethyl acetate, and n-butanol. Then, the part of the n-butanol extract was run by an HP-20 macroporous resin and eluted with water, 30% ethanol, and 50% ethanol. In this study, we assessed both the 30% ethanol and 50% ethanol extracts, as well as the initially obtained n-butanol extract. These extracts are intended to be enriched in flavonoid glycosides, catechins, and polar hydroxycinnamic acids, due to the successive liquid-liquid extractions and chromatography using the HP-20 resin.

### 4.2. Phytochemical Analysis

High-Performance Liquid Chromatography with Diode Array Detector (HPLC-DAD) was used to analyze the major constituents of *H. citrina* Baroni extracts as described elsewhere [5]. In brief, the *H. citrina* Baroni extracts were dissolved in methanol, filtered by a Nylaflo filter (nylon, 0.45 mM, Pall corporation, New York, NY, USA), poured into amber vials, and stored at 4 °C until further analysis. Separation was performed in a Purospher cartridge (250 × 4 mm, 5 mM, Merck, Darmstadt, Germany), data were recorded in the 200–600 mn range, and chromatograms were taken at 260, 280, 325, and 350 nm. Quantification was performed by the external standard method, using pure chlorogenic acid, epicatechin, and hyperoside (Sigma-Aldrich, St. Louis, MO, USA), at 325, 280, and 350 nm, respectively. Unknown hydroxycinnamic acids, catechins, and flavonols were quantified as chlorogenic, epicatechin, and hyperoside equivalents, respectively.

### 4.3. Nematode Strains and Maintenance

Animals were maintained in accordance with standard protocols [46], at a constant temperature of 20 °C in Petri dishes with nematode growth media (NGM) (17 g/L agar, 3 g/L NaCl, 2.5 g/L Bacto peptone, 1 mM MgSO_4_, 1 mM CaCl_2_, 25 mM phosphate buffer, 5 mg/mL cholesterol) and fed with OP50 *Escherichia coli*. Strains used in this work are listed in Supplementary Table S1.

Strains were synchronized by hypochlorite treatment (~2.6% NaClO and 0.5 M NaOH) for 7 min or as otherwise stated.

### 4.4. Nematode Treatments

Animals were treated with different concentrations of *H. citrina* Baroni or with selected compounds in liquid culture. Briefly, animals were synchronized by hypochlorite treatment and allowed to hatch overnight. L1 staged animals were then transferred to 96-well microtiter plates with the different concentrations of extract (0.125–1.000 mg/mL dissolved in 1% DMSO) or compounds (Quercetin, Isoquercitrin, Gallic Acid, and Chlorogenic Acid) (1–100 μM), S-media (NaCl 5.85 g/L, K_2_HPO_4_ 1 g/L, KH_2_PO_4_ 6 g/L, cholesterol (5 mg/mL in ethanol 100%) 1 mL/L, 10 mL/L trace metal, 10 mL/L potassium citrate dH_2_O), and OP50 *E. coli* with an OD = 0.9 or otherwise stated. Animals remained in the same solution for 4 days, at 20 °C and 180 RPM, until the day of the test. If assays were prolonged for more than 4 days, animals were washed and reincubated in a fresh S-media and extract or vehicle every day to discard progeny.

### 4.5. Extract Toxicity Assay

The toxicity of *H. citrina* Baroni extracts in vivo was determined using the wild-type N2 Bristol strain and the food clearance assay, in which the pattern of food consumption was used as a proxy for development delay or arrest as previously described [10,47]. Absorbance was measured daily at 595 nm using a Tecan Infinite M200 Pro plate reader (Männedorf, Switzerland).

### 4.6. Motility Assay

The motor phenotype of animals was scored using the motility assay as previously described in [10,47]. Bristol N2 Wild-type mutant ATXN3 (mtATXN3) and mutant Tau (mtTau)-expressing transgenic nematodes were grown in liquid culture as described in Section 4.5. On day 4, animals were transferred to unseeded NGM plates at 20 °C and allowed to dry for 45 min. Motility assays were performed as previously described [14,48]. In each assay, a minimum of 50 worms were assessed per condition, and triplicate assays (n = 3, total number of animals = 150) were performed.

### 4.7. Mutant Ataxin-3 Neuronal Aggregation Assay

Ataxin-3 neuronal aggregation was quantified by in vivo confocal microscopy as previously described [14,49]. Aggregation was automatically quantified using MeVisLab 3.6.1, as described by Teixeira-Castro and colleagues [49]. The parameters examined are as follows: total number of aggregates, number of aggregates/total area, and total area of aggregates/total area of the head. Triplicates were performed, with at least six animals per condition.

### 4.8. Transcriptional Reporter Strains

Synchronized L1 larvae of CL2166 [P*_gst-4_*:GFP], LD1171 [(P*_gcs-1_*:GFP + rol6(su1006)], SJ4100 [P*_hsp-6_::*GFP], SJ4005 [P*_hsp-4_*::GFP] and AM722 [P*_hsp-70_*::mCherry; myo-2p::CFP] strains were grown in liquid culture with 1 mg/mL of HCE30% or vehicle (1% Dimethilsulfoxide (DMSO)) until day 4 after hatching. The following positive controls of reporter gene activation were also included: the strains CL2166 and LD1171 were grown in a vehicle (1% DMSO) and incubated in 5-Hydroxy-1,4-naphthoquinone (juglone) (Sigma-Aldrich, St. Louis, MO, USA) at 150 µM for 1 h and allowed to recover at 20 °C for 3 h; the strain SJ4100 was treated with antimycin A, an oxidative phosphorylation inhibitor, at a final concentration of 5 µM during 24 h; for SJ4005, a 24 h treatment with the antibiotic tunicamycin, at a final concentration of 5 µg/mL was performed; AM722 animals were subjected to a 1 h heat-shock at 33 °C and allowed to recover for 7 h prior imaging. Fluorescence exposure time was adjusted to the DMSO 1% treatment. The fluorescence intensity of each worm was measured using Fiji (ImageJ, 1.54f) and normalized to the mean of the vehicle-treated worms. At least 10 animals per condition were scored in each experiment. Settings were adjusted to control conditions and used for the other conditions analyzed in each experiment.

All strains expressing the sqIs17 transgene [P_hlh-30_::hlh-30::GFP + rol-6(su1006)] were grown in liquid culture with 1 mg/mL of HCE or vehicle (1% DMSO) from day 1 to day 4 after hatching. Starvation was used as a positive control. For this, animals were transferred to unseeded plates for 30 min prior to imaging. Animals were considered to have nuclear translocation of HLH-30 if well-defined fluorescent nuclei were observed. At least 10 animals per condition were scored in each experiment.

All animals were anesthetized with sodium azide (2 mM) (Sigma, St. Louis, MO, USA) and mounted on 3% agarose pads. Excess azide was removed, and worms were covered with a cover slide. Brightfield and Fluorescence (Fluorescein Isothiocyanate (FITC) filter) images were acquired using an Olympus Widefield Upright Microscope BX61 (Olympus, Tokyo, Japan), at 4× and 10× magnification, or using an Olympus Widefield Inverted Microscope IX81 (Olympus, Tokyo, Japan) at 4× and 10× magnification.

### 4.9. Lipid Droplet Staining

Animals were grown and treated as previously described for 4 days after hatching. At day 4, animals were collected, washed three times in M9, and incubated with BODIPY 493/503 at 25 μM in M9 for 20 min with agitation. After incubation, worms were washed in M9 three times before being immobilized with 3 mM Levamisole (Sigma, St. Louis, MO, USA) on a 3% agarose pad and imaged using a confocal microscope Olympus FV1000 (Olympus, Tokyo, Japan).

### 4.10. Statistical Analysis

All statistical tests were performed using SPSS 27.0 (SPSS Inc., Chicago, IL, USA) or GraphPad Prism 9.5.0 (GraphPad Software Inc.; San Diego, CA, USA) and are reported in the Supplementary Materials. Graphs were drawn using GraphPad Prism 9.5.0. A 95% confidence interval was assumed for all tests. Food consumption curves, used as a proxy for toxicity, were analyzed by fitting a Non-linear regression for sigmoidal curves, and two parameters (IC50, HillSlope values) were used to compare the curves using the least squares model. Continuous variables are reported by the mean and standard error of the mean (SEM), while categorical variables are reported by proportions. The normality of distributions was assessed by the Shapiro-Wilk test and the homogeneity of variances by Brown–Forsythe test. For comparison of means of more than two groups, when all assumptions were met, one-way ANOVA was applied, followed by post-hoc bilateral Dunnett test or Tukey HSD. When normality was not assumed, the Kruskal–Wallis test was performed, and pairwise comparisons were corrected using the Bonferroni correction. Comparisons between two groups were performed using independent samples t-tests or Mann–Whitney U tests when normality was not assumed. Proportion analysis was performed using Pearson’s chi-square test, with the post hoc z-test for independent proportions with Bonferroni correction.

## Data Availability

The original contributions presented in this study are included in the article/Supplementary Materials. Further inquiries can be directed to the corresponding author.

## Supplementary Materials

The following supporting information can be downloaded at: https://www.mdpi.com/article/10.3390/ijms26094145/s1.

## Abbreviations

The following abbreviations are used in this manuscript:

ATXN3	Ataxin-3
CFP	Cyan Fluorescent Protein
DMSO	Dimethyl Sulfoxide
FITC	Fluorescein Isothiocyanate
GCS-1	Gamma-glutamyl Cysteine Synthetase 1
GFP	Green Fluorescent Protein
GST-4	Glutathione S-Transferase 4
HCE	*Hemerocallis citrina* ethanolic Extract
HCN	*Hemerocallis citrina* N-butanol extract
HLH	Helix-Loop-Helix
HPLC-DAD	High-Performance Liquid Chromatography with Diode Array Detector
HSR	Heat Shock Response
MAPT	Microtubule Associated Protein Tau
MiT/TFE	Microphthalmia/Transcription Factor E
MJD	Machado–Joseph Disease
mtATXN3	Mutant Ataxin-3
mtTau	Mutant Tau
NDs	Neurodegenerative Diseases
NGM	Nematode Growth Media
SCA3	Spinocerebellar Ataxia type 3
SEM	Standard Error of the Mean
SERT	Serotonin Transporter
TFEB	Transcription Factor EB
UPRER	Unfolded Protein Response of the Endoplasmic Reticulum
UPRmt	Unfolded Protein Response of the mitochondria
