# Supplementary material for: Therapeutic Effects of Hemerocallis citrina Baroni Extract on Animal Models of Neurodegenerative Diseases Through Serotonin and HLH-30/TFEB-Dependent Mechanisms
Source: Int J Mol Sci. 2025 Apr 27;26(9):4145. doi: 10.3390/ijms26094145 (PMC12071762; doi:10.3390/ijms26094145)
Supplement: Supplementary file 1 [file ijms-26-04145-s001.zip › ijms-3455009-supplementary.pdf]

Supplementary Material

Therapeutic Effects of *Hemerocallis citrina* Baroni Extract on Animal Models of Neurodegenerative Diseases Through Serotonin and HLH-30/TFEB-Dependent Mechanisms

Jorge H. Fernandes [1,2], Marta Daniela Costa [1,2], Daniela Vilasboas-Campos [1,2], Bruna Ferreira-Lomba [1,2],
Joana Pereira-Sousa [1,2], Qiong Wang [3], Andreia Teixeira-Castro [1,2], Xinmin Liu [4], Fengzhong Wang [3],
Alberto C. P. Dias [5] and Patrícia Maciel [1,2,*]

[1] Life and Health Sciences Research Institute (ICVS), School of Medicine, University of Minho,
4710-057 Braga, Portugal; id9966@alunos.uminho.pt (J.H.F.); martacosta@med.uminho.pt (M.D.C.);
id8808@alunos.uminho.pt (D.V.-C.); id11852@alunos.uminho.pt (B.F.-L.); b12998@med.uminho.pt (J.P.-S.);
accastro@med.uminho.pt (A.T.-C.)

[2] ICVS/3B's—PT Government Associate Laboratory, 4710-057 Braga, Portugal

[3] Institute of Food Science and Technology, Chinese Academy of Agricultural Sciences,
Beijing 100193, China; qiongwang623@foxmail.com (Q.W.); wangfengzhong@sina.com (F.W.)

[4] Institute of Drug Discovery Technology, Ningbo University, Ningbo 315211, China; liuxinmin@hotmail.com

[5] Centre of Molecular and Environmental Biology (CBMA), Department of Biology, University of Minho, 4710-057 Braga, Portugal; albertocpdias66@gmail.com

* Correspondence: pmaciel@med.uminho.pt

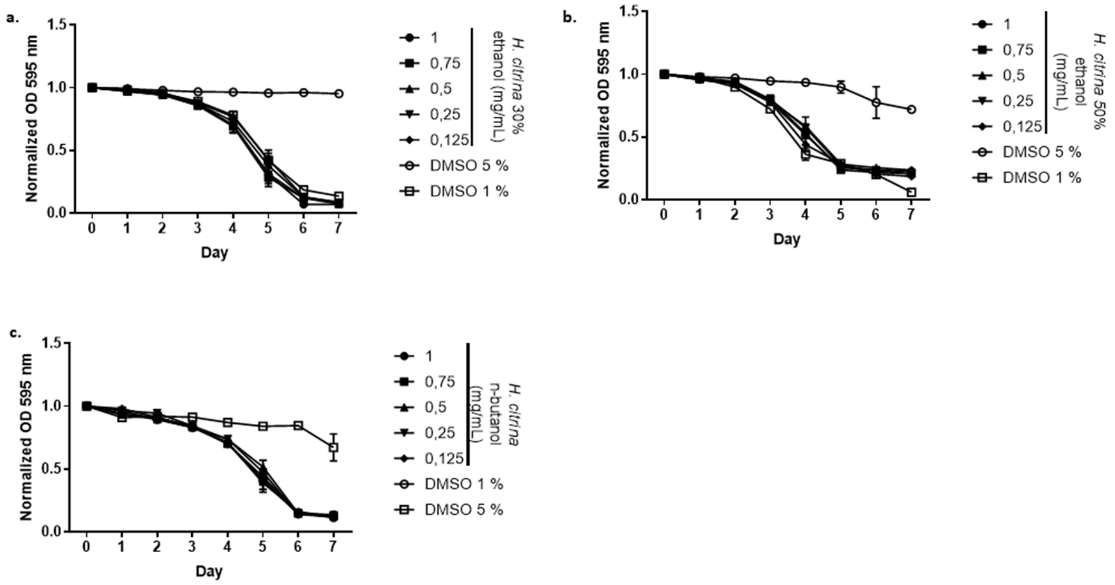

Supplementary Figure S1 - Treatment with *H. citrina* extracts did not provoke any toxic effect. Toxicity was evaluated using the food clearance assay, in *C. elegans*. Evaluation of the toxic effects of **a.** *H. citrina* 30% ethanol; **b.** *H. citrina* 50% ethanol; **c.** *H. citrina* n-butanol; The OD of *E. coli* was evaluated daily for each concentration and normalized for day 0 values. Statistical analysis revealed no significant differences between LogIC50 and HillSlope values of any treatment and DMSO 1% (drug vehicle, non-toxic). Graphic represented with Mean ± SEM.

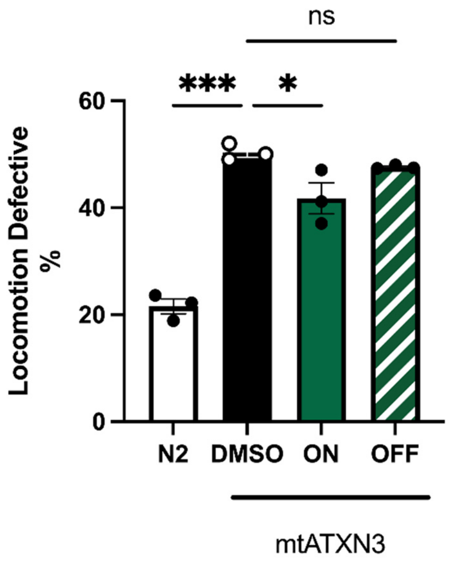

Supplementary Figure S2 – Six-day treatment with HCE30% improved the motor phenotype of mtATXN3 animals and is lost upon HCE withdrawn. Bars represent mean percentage of animals considered as locomotion defective in a motility assay ± SEM for 3 independent assays, at least 50 animals per condition, per assay (total number of animals = 150). $p \leq 0.05$ (*), $p \leq 0.001$ (***), no significant differences (n.s.).

.

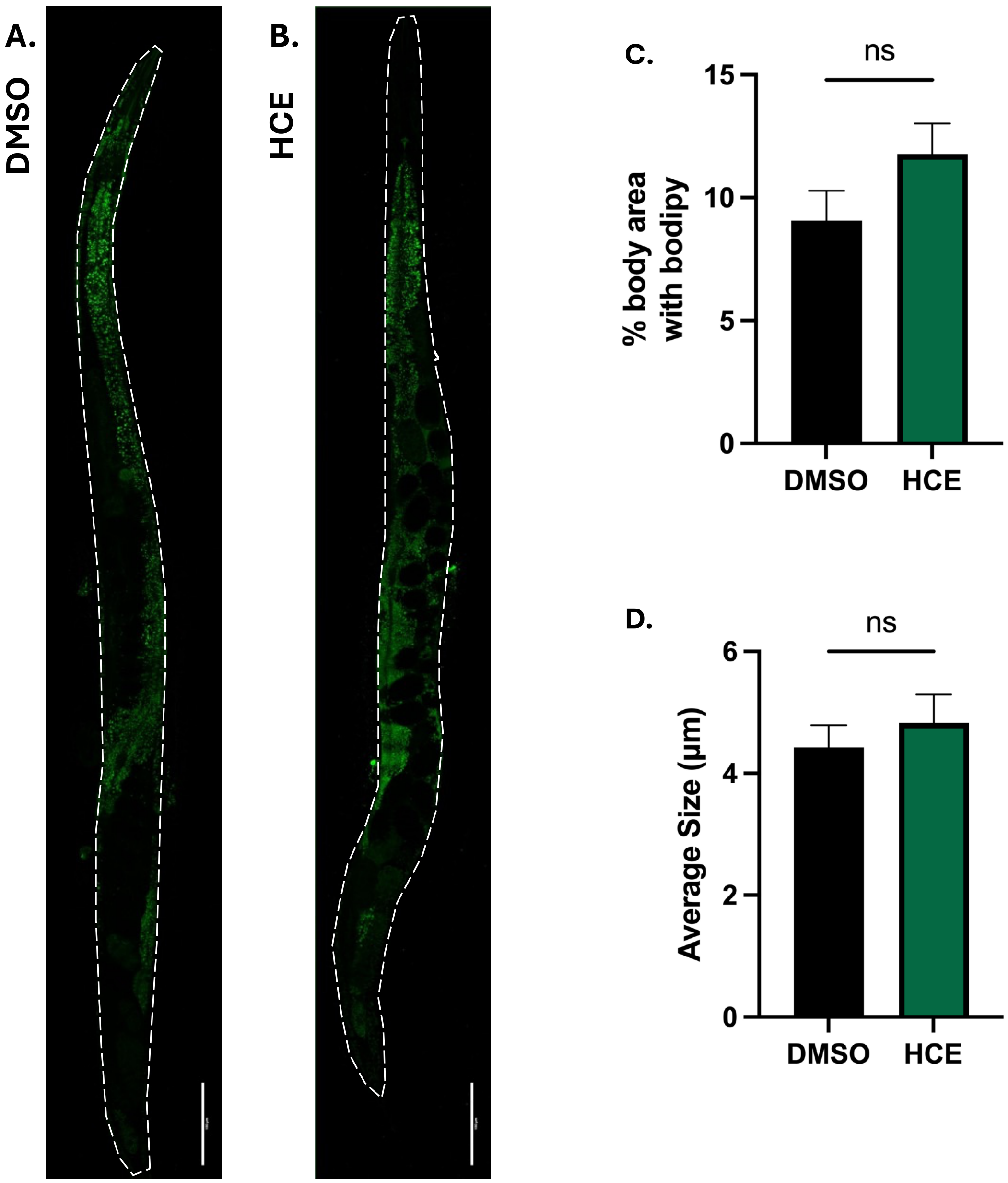

Supplementary Figure S3 – HCE treatment did not mobilize lipids from lipid droplets. Representative images of N2 worms treated with A. vehicle and B. HCE (1 mg/mL). Treatment with HCE did not change C. the percentage of body area stained with BODIPY 493/503 nor D. the average size of the lipid droplets. Bars represent mean ± SEM for 3 independent assays, at least 4 animals per condition, per assay (total number of animals = 13). No significant differences (ns).

Supplementary Table S1 – List of strains used in this paper.

Name	*Synonym*	*Genotype*
CK10	mtTau	bkIs10 [P_{aex-3}::hTau V337M + myo2p::GFP
AM685	mtATXN-3	rmls263[$P_{F25B3.3}$::AT3v1- 1q130::yfp]
MAC75	mtATXN3;*ser-1*	rmls263[$P_{F25B3.3}$::AT3v1- 1q130::yfp], *ser-1*(ok345) X;
DA1814	*ser-1*	*ser-1*(ok345) X;
MAC13	*ser-5;*mtATXN3	*ser-5* (tm2654) I; rmls263[$P_{F25B3.3}$::AT3v1- 1q130::yfp] II;
MAC38	mtATXN3 (WT *ser-5*)	*ser-5*(+)I; rmls263[$P_{F25B3.3}$::AT3v1- 1q130::yfp] II;
MAC17	mtATXN3;*ser-7*	rmls263[$P_{F25B3.3}$::AT3v1- 1q130::yfp] II; *ser-7*(tm1325) X;
DA2100	*ser-7*	*ser-7*(tm1325) X;
MAC39	mtATXN3 (WT *ser-7*)	rmls263[$P_{F25B3.3}$::AT3v1- 1q130::yfp] II; *ser-7* (+) X;
MAC72	mtATXN3;*ser-4*	*ser-4*(ok512) III;rmls263[$P_{F25B3.3}$::AT3v1-1q130::yfp];
AQ866	*ser-4*	*ser-4*(ok512) III;
MAC73	*mod-5;* mtATXN3	*mod-5*(n822) I;rmls263[$P_{F25B3.3}$::AT3v1-1q130::yfp];
MT8944	*mod-5*	*mod-5*(n822) I;
MAC171	mtATXN3;*dop-1*	rmls263[$P_{F25B3.3}$::AT3v1- 1q130::yfp] II; *dop-1*(vs101) X;
LX636	*dop-1*	*dop-1*(vs101) X;
MAC168	mtATXN3 (WT *dop-1*)	rmls263[$P_{F25B3.3}$::AT3v1- 1q130::yfp] II; *dop-1* (+) X;
MAC368	mtATXN3;*dop-3*	rmls263[$P_{F25B3.3}$::AT3v1- 1q130::yfp] II; *dop-3*(vs106) X;
LX703	*dop-3*	*dop-3*(vs106) X;
MAC367	mtATXN3 (WT *dop-3*)	rmls263[$P_{F25B3.3}$::AT3v1- 1q130::yfp] II; *dop-3* (+) X;
MAH240	HLH-30::GFP	sqIs17 [P_{hlh-30}::hlh-30::GFP + *rol-6*(su1006)];
LD1171	P*gcs*-1::GFP	ldIs3 [P*gcs*-1::GFP + rol-6(*su1006*)];
CL2166	P*gst-4*::GFP	dvIs19 [P*gst-4*::GFP::NLS] III;
AM722	P*hsp-70*::mCherry	rmIs288[P*hsp-70*::mCherry; P*myo-2*::CFP];
SJ4100	P*hsp-6*::GFP	zcIs13[P*hsp-6*::GFP] V;
SJ4005	P*hsp-4*::GFP	zcIs4[P*hsp-4*::GFP] V;
JIN1375	hlh-30	*hlh-30*(tm1978) IV
MAC437	mtATXN3 (WT *hlh-30)*	rmls263[$P_{F25B3.3}$::AT3v1-1q130::yfp] II
MAC438	mtATXN3;*hlh-30*	rmls263[$P_{F25B3.3}$::AT3v1-1q130::yfp] II; *hlh-30*(tm1978) IV
MAC439	mtTau (WT hlh-30)	bkIs10[P_{aex-3}::Tau–V337M; P*myo-2*::GFP]
MAC440	hlh-30;mtTau	hlh-30(tm1978) IV; bkIs10[P_{aex-3}::Tau–V337M; P*myo-2*::GFP]
MAC462	HLH-30::GFP;*ser-1*	sqIs17 [P_{hlh-30}::hlh-30::GFP + *rol-6*(su1006)]; *ser-1*(ok345) X

Supplementary Table S2 – HCE30% phytochemical composition.

Peak	*Compound*	*RT*	*UV Max*	*µg/mg*
E1	epicatechin	15.68	278	45
E2	epicatechin deriv	16.30	281, 321	30
Q	flavonoid (quercetin glucoside derivative)	16.79	255, 348	89
H1	hyroxycinnamic acid deriv	17,55	326	541
H2	hyroxycinnamic acid deriv	18,94	328	76
H3	hyroxycinnamic acid deriv	20.18	319	20
H4	hyroxycinnamic acid deriv	21.38	315	28
U	unknown	21.97	277, 335	-
H5	hyroxycinnamic acid deriv	23.37	327	14
H6	hyroxycinnamic acid deriv	24.38	327	10
	Total			*853*
